# Lost in a number: concealed heterogeneity within the sequential organ failure assessment (SOFA) score

**DOI:** 10.1186/s13054-023-04782-2

**Published:** 2024-01-02

**Authors:** Neville Dusaj, Eleni Papoutsi, Katherine L. Hoffman, Ilias I. Siempos, Edward James Schenck

**Affiliations:** 1 Tri-Institutional MD-PhD Program, Weill Cornell Medicine, Memorial Sloan Kettering Cancer Center, Rockefeller University, New York, NY USA; 2 First Department of Critical Care Medicine and Pulmonary Services, Evangelismos Hospital, National and Kapodistrian University of Athens Medical School, Athens, Greece; 3 Division of Biostatistics, Department of Population Health Sciences, Weill Cornell Medicine, New York, NY USA; 4 Division of Pulmonary and Critical Care Medicine, Department of Medicine, New York-Presbyterian Hospital-Weill Cornell Medical Center, Weill Cornell Medicine, 1300 York Avenue, Box 96, New York, NY 10065 USA

**Keywords:** Predictive enrichment, Prognostic enrichment, Heterogeneity of treatment effect, Multiple organ dysfunction scores, SOFA

**To the editor**:

Organ dysfunction scores [1] are used in critical care research to benchmark the risk of death in ICU populations and to explore potential heterogeneity of treatment effects in clinical trials. The SOFA score, an updatable organ dysfunction score made of six individual subscores, is used to define sepsis [2] and has been used in randomized clinical trials of sepsis and ARDS to define quantiles of risk to explore heterogeneity of the average treatment effect.

Implicit in the use of multiple organ dysfunction as a stratification method is the expectation that the approach will result in sub-populations that will be more homogeneous and share a similar prognosis. Unfortunately, this approach may not account for potential clinical and biologic heterogeneity. Such heterogeneity may dilute the predictive effect of grouping by a similar prognosis. Recent work has identified ICU subphenotypes using SOFA scores together with other biologic variables [3]. More simply, a single SOFA score number contains multiple combinations of disparate organ dysfunctions. For example, a score of 6 has 426 subscore combinations, and 12 has 1751. This heterogeneity may conceal varied pathobiology leading to a similar prognosis in critical illness. To illustrate this potential, we explored the heterogeneity within groups of patients sharing a single SOFA score.

We did a retrospective study using two data sources: a single-center ICU cohort, see supplemental methods for details, and the PETAL-ROSE multicenter randomized clinical trial of neuromuscular blockers for patients with ARDS [4]. We identified patients by Sepsis-3 criteria [2] in the ICU cohort, and then, we explored the heterogeneity within patients sharing a day 1 SOFA score of 6, 9, and 12. To validate this heterogeneity in a more specific disease we explored a population with a non-neurologic SOFA score of 9 in the ARDS clinical trial.

Within each strata of patients sharing the same total SOFA score, we performed a clustering analysis to identify subphenotypes. We compared SOFA subscores components, demographics and other baseline factors across clusters in each strata to identify underlying biologic differences. We then compared 28-day mortality and markers accounting for duration of organ failure support.

Within the ICU cohort population, there were 760, 469, 206 patients with a SOFA score of 6, 9 and 12, respectively. Three distinct subscore defined subphenotypes were seen in each group. For example, in the group with a SOFA of 9, higher cardiovascular failure scores, higher respiratory failure scores and higher mixed organ failure were seen as distinct clusters. Panel A of Fig. 1 displays the log2 fold change of each SOFA subscore in each subphenotype. Similar findings were seen in the SOFA 6 and SOFA 12 strata with different subscore distributions. Consistently, three distinct clusters were seen in the clinical trial population. Details of the total populations and each subphenotype can be found in the supplement, Additional file 1: Tables S1–S4, and Figures S1–S4.

**Fig. 1:**
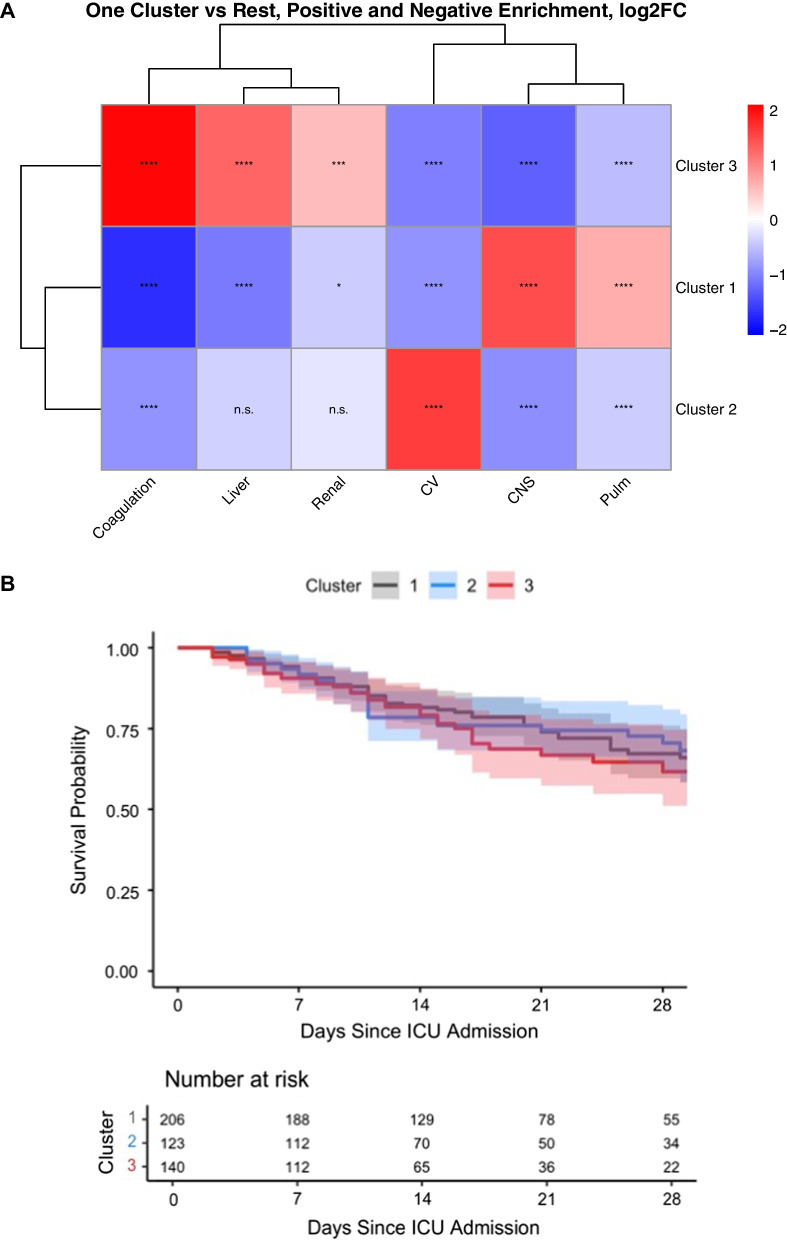
**A** Heat map of differing clusters with log2 fold differences in SOFA subscores. Color intensity corresponds to log2 fold changes, and number of * correspond to statistical significance. Abbreviations: CV Cardiovascular, CNS Central Nervous System, **B** Kaplan–Meier plot comparing survival time between clusters

In the SOFA 9 strata in the ICU cohort, patients in the cardiovascular failure cluster were older, more likely to be women, to have a blood stream infection, and have septic shock compared to the two other cohorts. Patients in the respiratory failure cohort had more comorbidities and were more likely to have pneumonia. Patients in the mixed group were younger and were more likely to have immunosuppression compared to patients in other subphenotypes. Differential clusters were seen in the other SOFA score strata and in the ARDS clinical trial, Additional file 1: Table S4 and Figures S5, S6. All SOFA score strata in each case shared a similar prognosis, Additional file 1: Tables S1–S4; however, individual organ dysfunction durations and clinical characteristics were different.

In two independent cohorts, we identified distinct clusters of patients within different SOFA scores each with a similar prognosis but with markedly different clinical characteristics. This analysis displays the hidden heterogeneity within multiple organ dysfunction scores despite accuracy in identifying similar outcomes. This study compliments work that established that an organ dysfunction scores’ validity is a function of the scores’ uniformity of fit to the population under study [5] and highlights that predictive enrichment may not be achieved with methods that are prognostically valid.

Strengths include the simple design and inclusion of a broad range of patients from two distinct data sources reflecting a range of ICU patients. Limitations include using the relatively inclusive definition of sepsis from a single academic center with a high severity of disease. Moreover, the use of electronic health records may lead to missingness and confounding regarding neurologic injury. However, we confirmed similar findings in a more restrictive ARDS clinical trial population with manually extracted data. We chose to explore the hidden heterogeneity in the simple SOFA score, a more complicated risk prediction scoring system would by definition conceal more heterogeneity. This analysis supports explicit hypothesis-driven predictive enrichment in the design of clinical trials. A number is not a surrogate for clinical homogeneity.

### Supplementary Information

**Additional file 1**. Supplemental Methods, Tables and Figures.

## Data Availability

The datasets analyzed during the current study are available from the corresponding author on reasonable request.

## Abbreviations

APACHE: Acute physiology and chronic health valuation
ICU: Intensive care unit
SOFA: Sequential organ failure assessment
