# Supplementary material for: Lost in a number: concealed heterogeneity within the sequential organ failure assessment (SOFA) score
Source: Crit Care. 2024 Jan 2;28:6. doi: 10.1186/s13054-023-04782-2 (PMC10759548; doi:10.1186/s13054-023-04782-2)
Supplement: Supplementary file 1 — Additional file 1. Supplemental Methods, Tables and Figures. [file 13054_2023_4782_MOESM1_ESM.docx]

Lost in a number: Concealed heterogeneity within the Sequential Organ Failure Assessment (SOFA) Score

Neville Dusaj^1^, Eleni Papoutsi^2^, Katherine L Hoffman^3^, Ilias I Siempos^2,4^, Edward J Schenck^4^

Affiliations:

1 Tri-Institutional MD-PhD Program, Weill Cornell Medicine, Rockefeller University, Memorial Sloan Kettering Cancer Center, New York, NY, United States of America

2 First Department of Critical Care Medicine and Pulmonary Services, Evangelismos Hospital, National and Kapodistrian University of Athens Medical School, Athens, Greece

3 Division of Biostatistics, Department of Population Health Sciences, Weill Cornell Medicine, New York, New York, United State of America

4 Division of Pulmonary and Critical Care Medicine, Department of Medicine, New York-Presbyterian Hospital-Weill Cornell Medical Center, Weill Cornell Medicine, New York, New York, United States of America

Supplemental Methods:

The intensive care unit (ICU) cohort included adult patients admitted to an ICU from a single Quaternary Hospital located in New York City^1^. In the ICU cohort, Sequential Organ Failure Assessment (SOFA) was calculated using previously identified methodology^2^. In brief, each SOFA subscore was calculated as the worst value of the first 24-hour period following admission to the ICU. For the respiratory subscore, we substituted a SpO_2_ when a PaO_2_ was not available using the linear method proposed by Rice et al^3^. For the cardiovascular subscore we calculated a norepinephrine equivalency as suggested by the randomized clinical trial for the approval of angiotensin 2^4^. The neurologic subscore was calculated by the worst GCS of the day regardless of the presence of sedation. Missing values were considered normal following the guidance of Sepsis-3^5^. We explored the heterogeneity within patients exhibiting a day 1 of ICU SOFA score of 6, 9, and 12^6^. Within the clinical trial population we explored heterogeneity within a strata with a non-neurologic SOFA of 9^7^. Within the ICU cohort of septic patients with a total SOFA score of 6, 9 and 12 we performed a clustering analysis to identify subphenotypes. We used the clusGap function of the R cluster package (v2.1.6) to determine the optimal number of clusters. We then used the kmeans function from the R stats package (v4.2.1) to assign each patient one of three clusters using the Hartigan and Wong algorithm, allowing for 10 random sets of starting points for convergence.

We compared SOFA subscores components across the three clusters to identify underlying biologic differences. For each cluster, we calculated the log2-fold-change of each of the 6 SOFA subscores for patients in versus out of the cluster. Nonparametric Wilcoxon signed-rank test (wilcox_test in rstatix package, v0.7.0) was used to calculate significance in difference between patients in and out of cluster, and these P-values were adjusted using the Bonferroni correction. A heatmap of log2-fold-change differences was generated to visualize associated organ systems as likely underlying drivers of patients’ illnesses. We descriptively examined demographic and other baseline characteristics using medians, interquartile ranges, sample size and percentages across clusters. We then examined 28 day mortality using Kaplan Meier curves and a log-rank test.

To further validate our findings in another dataset, we looked towards the ROSE trial^7^ for additional patients. As above we isolated those patients with an initial SOFA score of 9 excluding the neurologic subscore. The NbClust function from the NbClust package (v3.01) was used to determine the optimal number of clusters. Next, the kmeans function from the R stats package (v4.2.1) to assign each patient one of three clusters using the Hartigan and Wong algorithm. Finally, as above, log2-fold-change of each of the 6 SOFA subscores for patients in versus out of the cluster were calculated and compared using a Wilcoxon-signed-rank test. The results were visualized using a heatmap generated by the pheatmap package.

Supplemental Results:

| Supplemental Table 1. ICU Cohort characteristics and outcomes for patients with initial SOFA 6 | | | | | |
| --- | --- | --- | --- | --- | --- |
| ****CHARACTERISTIC**** | **Overall**, N = 760*^1^* | **1**, N = 201*^1^* | **2**, N = 264*^1^* | **3**, N = 295*^1^* | **p-value***^2^* |
| Age | 73 (61, 86) | 67 (57, 79) | 77 (66, 87) | 73 (62, 86) | <0.001 |
| Female | 354 (47%) | 87 (43%) | 113 (43%) | 154 (52%) | 0.046 |
| BMI | 26 (22, 31) | 26 (23, 31) | 26 (22, 31) | 25 (22, 31) | 0.8 |
| Sepsis severity |  |  |  |  | 0.063 |
| Not septic | 0 (0%) | 0 (0%) | 0 (0%) | 0 (0%) |  |
| Septic | 720 (95%) | 193 (96%) | 243 (92%) | 284 (96%) |  |
| Septic shock | 40 (5.3%) | 8 (4.0%) | 21 (8.0%) | 11 (3.7%) |  |
| ICU wing |  |  |  |  | <0.001 |
| Medical | 543 (71%) | 160 (80%) | 214 (81%) | 169 (57%) |  |
| Surgical | 217 (29%) | 41 (20%) | 50 (19%) | 126 (43%) |  |
| Total Elixhauser comorbidities | 5.00 (3.00, 7.00) | 5.00 (3.00, 7.00) | 6.00 (4.00, 8.00) | 4.00 (3.00, 6.00) | <0.001 |
| Immunosuppressed |  |  |  |  | <0.001 |
| Immunosuppressed | 304 (40%) | 108 (54%) | 104 (39%) | 92 (31%) |  |
| Not immunosuppressed | 417 (55%) | 83 (41%) | 137 (52%) | 197 (67%) |  |
| Solid organ transplant | 39 (5.1%) | 10 (5.0%) | 23 (8.7%) | 6 (2.0%) |  |
| Laboratory based immunosuppression |  |  |  |  |  |
| Neutropenia | 25 (3.3%) | 22 (11%) | 2 (0.8%) | 1 (0.3%) | <0.001 |
| SOFA Subscores |  |  |  |  |  |
| CV | 1.00 (1.00, 1.00) | 1.00 (0.00, 1.00) | 1.00 (1.00, 2.00) | 1.00 (1.00, 1.00) | <0.001 |
| Neurologic | 1.00 (0.00, 2.00) | 0.00 (0.00, 0.00) | 0.00 (0.00, 1.00) | 2.00 (2.00, 2.00) | <0.001 |
| Liver | 0.00 (0.00, 1.00) | 1.00 (0.00, 2.00) | 0.00 (0.00, 1.00) | 0.00 (0.00, 1.00) | <0.001 |
| Pulmonary | 2.00 (1.00, 2.00) | 1.00 (0.00, 2.00) | 1.00 (1.00, 2.00) | 2.00 (2.00, 3.00) | <0.001 |
| Renal | 1.00 (0.00, 2.00) | 0.00 (0.00, 1.00) | 2.00 (1.00, 2.00) | 0.00 (0.00, 1.00) | <0.001 |
| Coagulation | 0.00 (0.00, 1.75) | 2.00 (2.00, 3.00) | 0.00 (0.00, 1.00) | 0.00 (0.00, 0.50) | <0.001 |
| Source of infection |  |  |  |  |  |
| CNS | 8 (1.1%) | 3 (1.5%) | 2 (0.8%) | 3 (1.0%) | 0.8 |
| Intra-abdominal | 134 (18%) | 51 (25%) | 45 (17%) | 38 (13%) | 0.002 |
| Pneumonia | 294 (39%) | 63 (31%) | 102 (39%) | 129 (44%) | 0.021 |
| Septicemia/bacteremia | 315 (41%) | 92 (46%) | 131 (50%) | 92 (31%) | <0.001 |
| Soft tissue | 52 (6.8%) | 8 (4.0%) | 23 (8.7%) | 21 (7.1%) | 0.13 |
| Genitourinary | 178 (23%) | 37 (18%) | 79 (30%) | 62 (21%) | 0.007 |
| Died in 28d | 104 (14%) | 28 (14%) | 38 (14%) | 38 (13%) | 0.9 |
| Duration of mechanical ventilation | 0.0 (0.0, 2.0) | 0.0 (0.0, 0.0) | 0.0 (0.0, 0.0) | 1.0 (0.0, 6.0) | <0.001 |
| Duration of vasopressors | 0.00 (0.00, 2.00) | 0.00 (0.00, 0.00) | 0.00 (0.00, 3.00) | 0.00 (0.00, 1.00 | <0.001 |
| *^1^* median (iqr); n (%)  , BMI: body mass index, CNS: central nervous system, ICU: intensive care unit, SOFA: sequential organ failure assessment | | | | | |

Supplemental Figure 1. Heat Map of differing clusters with log2 fold differences in SOFA subscores for patients with initial SOFA 6. Color intensity corresponds to log2 fold changes, and number of * correspond to statistical significance. Abbreviations: CV Cardiovascular, CNS Central Nervous System


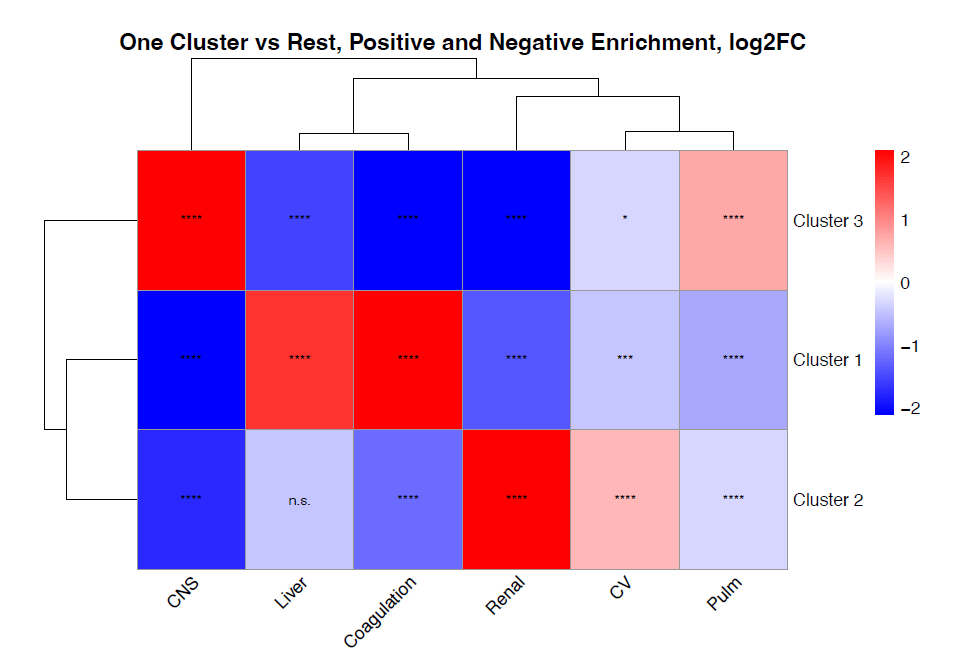


Supplemental Figure 2. Kaplan Meier plot comparing survival time between clusters of patients with initial SOFA 6.


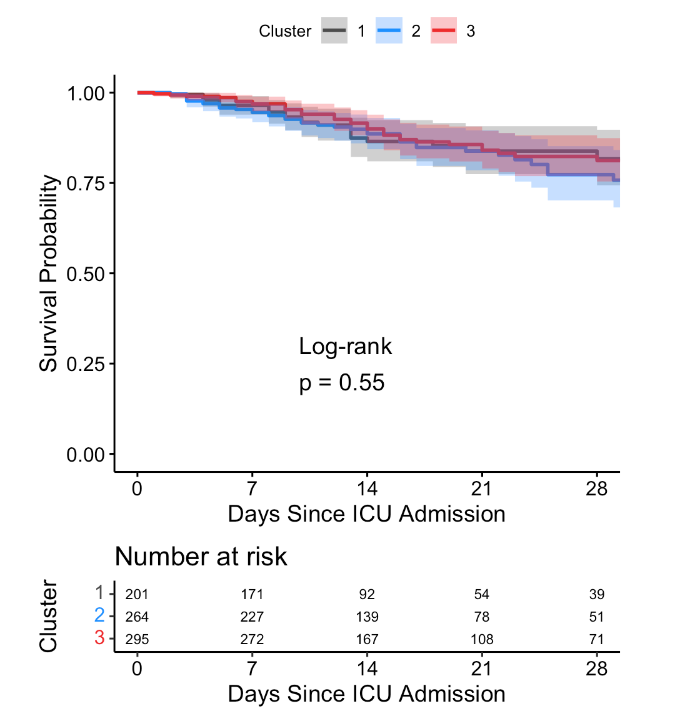


| Supplemental Table 2. ICU Cohort characteristics and outcomes SOFA 9 | | | | | |
| --- | --- | --- | --- | --- | --- |
| Characteristic | **Overall**, N = 469*^1^* | **1**, N = 206*^1^* | **2**, N = 123*^1^* | **3**, N = 140*^1^* | **p.value** |
| Age | 72 (61, 83) | 72 (59, 83) | 75 (66, 86) | 70 (61, 79) | 0.054 |
| Female | 183 (39%) | 77 (37%) | 54 (44%) | 52 (37%) | 0.4 |
| BMI | 27 (23, 32) | 28 (24, 34) | 26 (22, 30) | 27 (23, 31) | 0.027 |
| Sepsis severity |  |  |  |  | <0.001 |
| Not septic | 0 (0%) | 0 (0%) | 0 (0%) | 0 (0%) |  |
| Septic | 369 (79%) | 181 (88%) | 56 (46%) | 132 (94%) |  |
| Septic shock | 100 (21%) | 25 (12%) | 67 (54%) | 8 (5.7%) |  |
| ICU wing |  |  |  |  | 0.11 |
| Medical | 338 (72%) | 141 (68%) | 87 (71%) | 110 (79%) |  |
| Surgical | 131 (28%) | 65 (32%) | 36 (29%) | 30 (21%) |  |
| Total elixhauser comorbidities | 5.00 (3.00, 7.00) | 5.50 (4.00, 7.00) | 5.00 (3.00, 7.00) | 5.00 (3.00, 7.00) | 0.6 |
| Immunosuppressed |  |  |  |  | <0.001 |
| Immunosuppressed | 165 (35%) | 52 (25%) | 44 (36%) | 69 (49%) |  |
| Not immunosuppressed | 271 (58%) | 147 (71%) | 72 (59%) | 52 (37%) |  |
| Solid organ transplant | 33 (7.0%) | 7 (3.4%) | 7 (5.7%) | 19 (14%) |  |
| Laboratory based immunosupression |  |  |  |  |  |
| Neutropenia | 21 (4.5%) | 2 (1.0%) | 8 (6.5%) | 11 (7.9%) | 0.004 |
| SOFA Subscores |  |  |  |  |  |
| Cardiovascular | 1.00 (1.00, 3.00) | 1.00 (1.00, 2.00) | 4.00 (3.00, 4.00) | 1.00 (1.00, 1.00) | <0.001 |
| Neurologic | 2.00 (1.00, 3.00) | 3.00 (2.00, 4.00) | 1.00 (0.00, 2.00) | 1.00 (0.00, 2.00) | <0.001 |
| Liver | 1.00 (0.00, 2.00) | 0.00 (0.00, 1.00) | 0.00 (0.00, 1.00) | 2.00 (1.00, 2.00) | <0.001 |
| Pulmonary | 2.00 (2.00, 3.00) | 3.00 (2.00, 4.00) | 2.00 (1.00, 2.00) | 2.00 (1.00, 2.00) | <0.001 |
| Renal | 1.00 (0.00, 2.00) | 1.00 (0.00, 2.00) | 1.00 (0.00, 2.00) | 1.00 (0.00, 2.00) | 0.001 |
| Coagulation | 1.00 (0.00, 2.00) | 0.00 (0.00, 1.00) | 0.00 (0.00, 1.00) | 2.00 (2.00, 3.00) | <0.001 |
| Source of infection |  |  |  |  |  |
| CNS | 8 (1.7%) | 6 (2.9%) | 2 (1.6%) | 0 (0%) | 0.11 |
| Intra-abdominal | 89 (19%) | 23 (11%) | 34 (28%) | 32 (23%) | <0.001 |
| Pneumonia | 210 (45%) | 113 (55%) | 48 (39%) | 49 (35%) | <0.001 |
| Bacteremia | 258 (55%) | 99 (48%) | 79 (64%) | 80 (57%) | 0.014 |
| Soft tissue | 38 (8.1%) | 18 (8.7%) | 11 (8.9%) | 9 (6.4%) | 0.7 |
| Genitourinary | 100 (21%) | 40 (19%) | 35 (28%) | 25 (18%) | 0.075 |
| Died in 28 days | 120 (26%) | 55 (27%) | 31 (25%) | 34 (24%) | 0.9 |
| Duration of mechanical ventilation | 1.0 (0.0, 6.0) | 3.5 (0.0, 9.0) | 0.0 (0.0, 4.0) | 0.0 (0.0, 2.0) | <0.001 |
| Duration of vasopressors, Mean (SD) | 1.0 (0.0, 4.0) | 0.0 (0.0, 3.0) | 4.0 (2.0, 6.5) | 0.0 (0.0, 1.0) | <0.001 |
| *^1^* median (iqr); n (%)  , BMI: body mass index, CNS: central nervous system, ICU: intensive care unit, SOFA: sequential organ failure assessment | | | | | |

| Supplemental Table 3. ICU Cohort characteristics and outcomes SOFA 12 | | | | | |
| --- | --- | --- | --- | --- | --- |
| **Characteristic** | **Overall**, N = 206*^1^* | **1**, N = 57*^1^* | **2**, N = 94*^1^* | **3**, N = 55*^1^* | **p-value***^2^* |
| Age | 72 (63, 81) | 70 (64, 80) | 76 (66, 84) | 69 (59, 79) | 0.035 |
| Female | 75 (36%) | 19 (33%) | 34 (36%) | 22 (40%) | 0.8 |
| BMI | 27.9 (23.7, 31.3) | 27.9 (24.0, 30.5) | 27.5 (23.6, 30.7) | 29.4 (24.4, 33.2) | 0.4 |
| Sepsis Severity |  |  |  |  | <0.001 |
| Not Septic | 0 (0%) | 0 (0%) | 0 (0%) | 0 (0%) |  |
| Septic | 103 (50%) | 33 (58%) | 19 (20%) | 51 (93%) |  |
| Septic Shock | 103 (50%) | 24 (42%) | 75 (80%) | 4 (7.3%) |  |
| ICU Wing |  |  |  |  | 0.5 |
| Medical | 146 (71%) | 43 (75%) | 67 (71%) | 36 (65%) |  |
| Surgical | 60 (29%) | 14 (25%) | 27 (29%) | 19 (35%) |  |
| Total Elixhauser Comorbidities | 5.50 (4.00, 7.00) | 6.00 (5.00, 7.00) | 5.00 (3.00, 6.00) | 6.00 (3.50, 7.50) | 0.042 |
| Immunosuppressed |  |  |  |  | <0.001 |
| Immunosuppressed | 74 (36%) | 28 (49%) | 27 (29%) | 19 (35%) |  |
| Not Immunosuppressed | 116 (56%) | 22 (39%) | 66 (70%) | 28 (51%) |  |
| Solid organ transplant | 16 (7.8%) | 7 (12%) | 1 (1.1%) | 8 (15%) |  |
| Laboratory based immunosupression |  |  |  |  |  |
| Neutropenia | 12 (5.8%) | 7 (12%) | 1 (1.1%) | 4 (7.3%) | 0.006 |
| SOFA Subscores |  |  |  |  |  |
| Cardiovascular | 3.00 (1.00, 4.00) | 3.00 (1.00, 4.00) | 4.00 (4.00, 4.00) | 1.00 (1.00, 1.00) | <0.001 |
| Neurologic | 2.00 (2.00, 4.00) | 1.50 (1.00, 2.00) | 3.00 (2.00, 4.00) | 4.00 (3.00, 4.00) | <0.001 |
| Liver | 1.00 (0.00, 2.00) | 2.00 (2.00, 2.00) | 0.00 (0.00, 1.00) | 1.00 (0.00, 2.00) | <0.001 |
| Pulmonary | 3.00 (2.00, 3.75) | 2.00 (1.00, 2.00) | 3.00 (2.00, 4.00) | 3.00 (3.00, 4.00) | <0.001 |
| Renal | 1.00 (1.00, 2.00) | 2.00 (1.00, 2.00) | 1.00 (1.00, 2.00) | 1.00 (1.00, 2.00) | 0.029 |
| Coagulation | 1.00 (0.00, 2.00) | 3.00 (2.00, 3.00) | 0.00 (0.00, 1.00) | 2.00 (1.00, 2.50) | <0.001 |
| Source of infection |  |  |  |  |  |
| CNS | 2 (1.0%) | 0 (0%) | 1 (1.1%) | 1 (1.8%) | 0.7 |
| Intra-abdominal | 37 (18%) | 13 (23%) | 14 (15%) | 10 (18%) | 0.5 |
| Pneumonia | 103 (50%) | 26 (46%) | 46 (49%) | 31 (56%) | 0.5 |
| Septicemia/Bacteremia | 125 (61%) | 39 (68%) | 58 (62%) | 28 (51%) | 0.2 |
| Soft Tissue | 4 (1.9%) | 1 (1.8%) | 3 (3.2%) | 0 (0%) | 0.6 |
| Genitourinary | 45 (22%) | 16 (28%) | 17 (18%) | 12 (22%) | 0.4 |
| Death in 28 Days | 68 (33%) | 16 (28%) | 35 (37%) | 17 (31%) | 0.5 |
| Duration of mechanical ventilation | 3 (0, 8) | 1 (0, 3) | 3 (0, 8) | 6 (2, 13) | <0.001 |
| Duration of Vasopressors | 3.0 (1.0, 6.0) | 3.0 (1.0, 6.0) | 5.0 (3.0, 7.0) | 0.0 (0.0, 2.0) | <0.001 |
| *^1^* median (iqr); n (%)  , BMI: body mass index, CNS: central nervous system, ICU: intensive care unit, SOFA: sequential organ failure assessment | | | | | |

Supplemental Figure 3. Heat Map of differing clusters with log2 fold differences in SOFA subscores for patients with initial SOFA 12. Color intensity corresponds to log2 fold changes, and number of * correspond to statistical significance. Abbreviations: CV Cardiovascular, CNS Central Nervous System


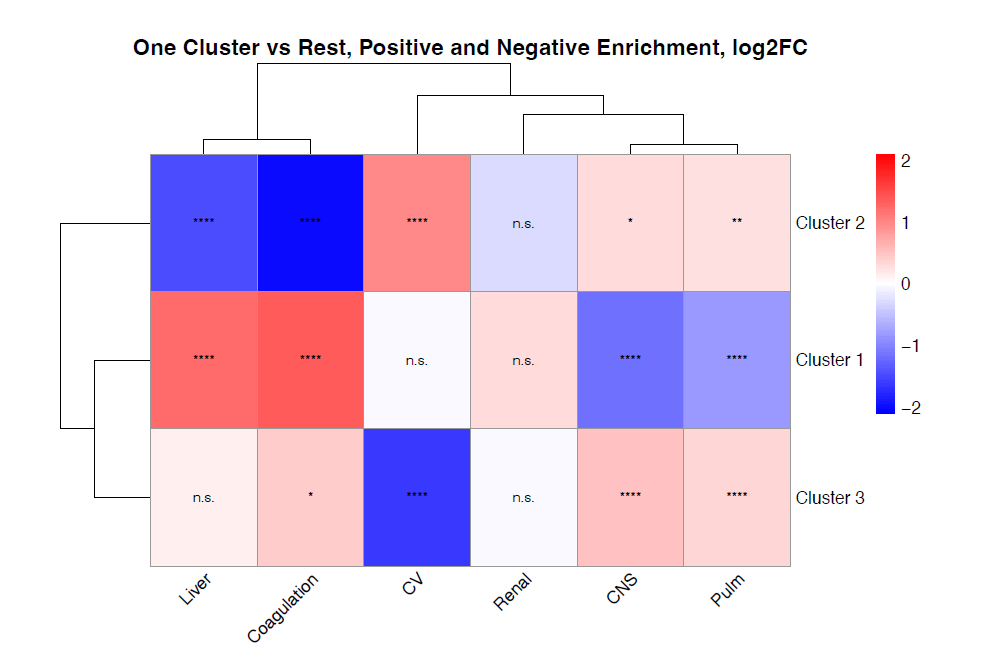


| Supplemental Figure 4. Kaplan Meier plot comparing survival time between clusters of patients with initial SOFA 12.  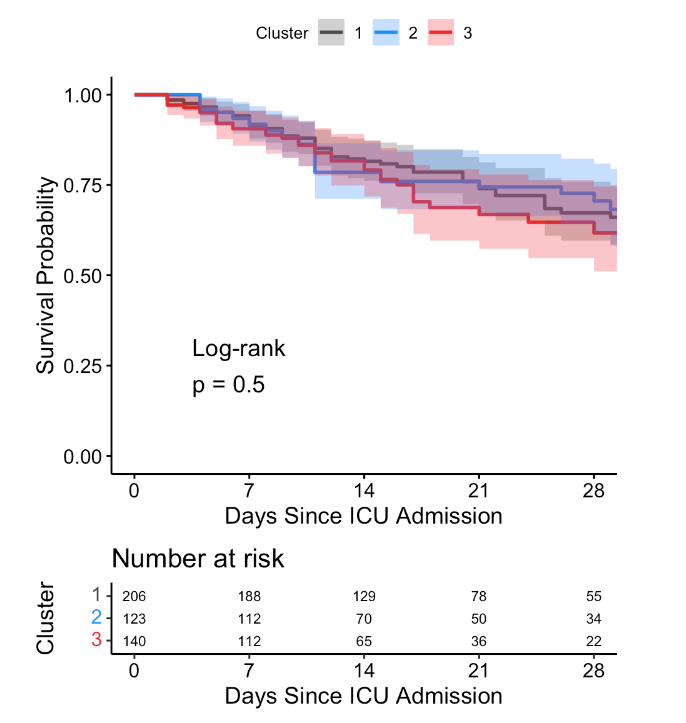  Supplemental Table 4. ROSE ARDS Clinical Trial cohort characteristics and outcomes | | | | | |
| --- | --- | --- | --- | --- | --- |
| **CHARACTERISTIC** | **Overall, N=86***^1^* | **1, N=20***^1^* | **2, N=50***^1^* | **3, N=16***^1^* | **p value** |
| **Age** | 59 (48, 69) | 56 (42, 68) | 61 (50, 70) | 58 (41, 67) | 0.617 |
| **Female** | 38 (44%) | 10 (50%) | 19 (38%) | 9 (56%) | 0.369 |
| **BMI** | 29 (23, 35) | 28 (25, 32) | 30 (24, 35) | 24 (22, 36) | 0.652 |
| **Race** |  |  |  |  | 0.820 |
| **White, not of Hispanic origin** | 53 (68%) | 10 (60%) | 32 (71%) | 11 (69%) |  |
| **Black, not of Hispanic origin** | 16 (21%) | 5 (29%) | 8 (18%) | 3 (19%) |  |
| **Hispanic or Latino** | 7 (9%) | 2 (12%) | 3 (7%) | 2 (13%) |  |
| **Other** | 2 (3%) | 0 (0%) | 2 (4%) | 0 (0%) |  |
| **Comorbidities** |  |  |  |  |  |
| **AIDS** | 2 (2%) | 0 (0%) | 2 (4%) | 0 (0%) | 1.0 |
| **Leukemia** | 3 (4%) | 0 (0%) | 1 (2%) | 2 (14%) | 0.111 |
| **Non-Hodgkin Lymphoma** | 1 (1%) | 0 (0%) | 1 (2%) | 0 (0%) | 1.0 |
| **Solid tumor with metastasis** | 4 (5%) | 0 (0%) | 3 (6%) | 1 (7%) | 0.619 |
| **Immunosuppression** | 11 (13%) | 2 (10%) | 6 (12%) | 3 (21%) | 0.656 |
| **Type of hospital admission** |  |  |  |  | 0.465 |
| **Medical** | 75 (87%) | 18 (90%) | 43 (86%) | 14 (88%) |  |
| **Surgical** | 5 (6%) | 0 (0%) | 3 (6%) | 2 (13%) |  |
| **Other** | 6 (7%) | 2 (10%) | 4 (8%) | 0 (0%) |  |
| **Primary risk factor of ARDS** |  |  |  |  |  |
| **Pneumonia** | 47 (55%) | 12 (60%) | 28 (56%) | 7 (44%) | 0.596 |
| **Sepsis** | 10 (12%) | 2 (10%) | 4 (8%) | 4 (25%) | 0.231 |
| **Aspiration** | 16 (19%) | 2 (10%) | 12 (24%) | 2 (13%) | 0.411 |
| **Trauma** | 5 (6%) | 3 (15%) | 2 (4%) | 0 (0%) | 0.155 |
| **Source of infection for sepsis** |  |  |  |  | 1.0 |
| **Thorax** | 7 (70%) | 2 (100%) | 2 (50%) | 3 (75%) |  |
| **Abdominal** | 1 (10%) | 0 (0%) | 1 (25%) | 0 (0%) |  |
| **Skin or soft tissue** | 1 (10%) | 0 (0%) | 0 (0%) | 1 (6%) |  |
| **SOFA Subscores** |  |  |  |  |  |
| **Cardiovascular** | 3.00 (1.00, 4.00) | 1.00 (1.00, 3.00) | 4.00 (4.00, 4.00) | 1.00 (0.00, 1.00) | <0.001 |
| **Liver** | 0.00 (0.00, 1.00) | 0.00 (0.00, 0.00) | 0.00 (0.00, 0.00) | 2.00 (0.25, 2.00) | <0.001 |
| **Pulmonary** | 3.00 (3.00, 4.00) | 3.00 (3.00, 4.00) | 3.00 (3.00, 4.00) | 3.00 (3.00, 3.75) | 0.204 |
| **Renal** | 1.00 (0.00, 2.25) | 3.00 (3.00, 4.00) | 1.00 (0.00, 1.00) | 1.00 (0.00, 1.75) | <0.001 |
| **Coagulation** | 1.00 (0.00, 2.00) | 0.00 (0.00, 1.00) | 0.00 (0.00, 1.00) | 2.50 (2.00, 3.00) | <0.001 |
| **Outcomes** |  |  |  |  |  |
| **28-day mortality** | 27 (31%) | 5 (25%) | 15 (30%) | 7 (44%) | 0.459 |
| **Organ failure-free days** | 14 (0, 25) | 8 (0, 21) | 22 (1, 26) | 3 (0, 14) | 0.011 |
| **Ventilator-free days** | 5 (0, 20) | 5 (0, 20) | 15 (0, 20) | 0 (0, 15) | 0.132 |
| **ICU-free days** | 9 (0, 16) | 7 (0, 20) | 10 (0, 18) | 1 (0, 12) | 0.148 |
|  |  |  |  |  |  |

Supplemental Figure 5. Heat Map of differing clusters with log2 fold differences in SOFA subscores for ARDS Clinical Trial patients with initial SOFA 9. Color intensity corresponds to log2 fold changes, and number of * correspond to statistical significance. Abbreviations: CV Cardiovascular, CNS Central Nervous System


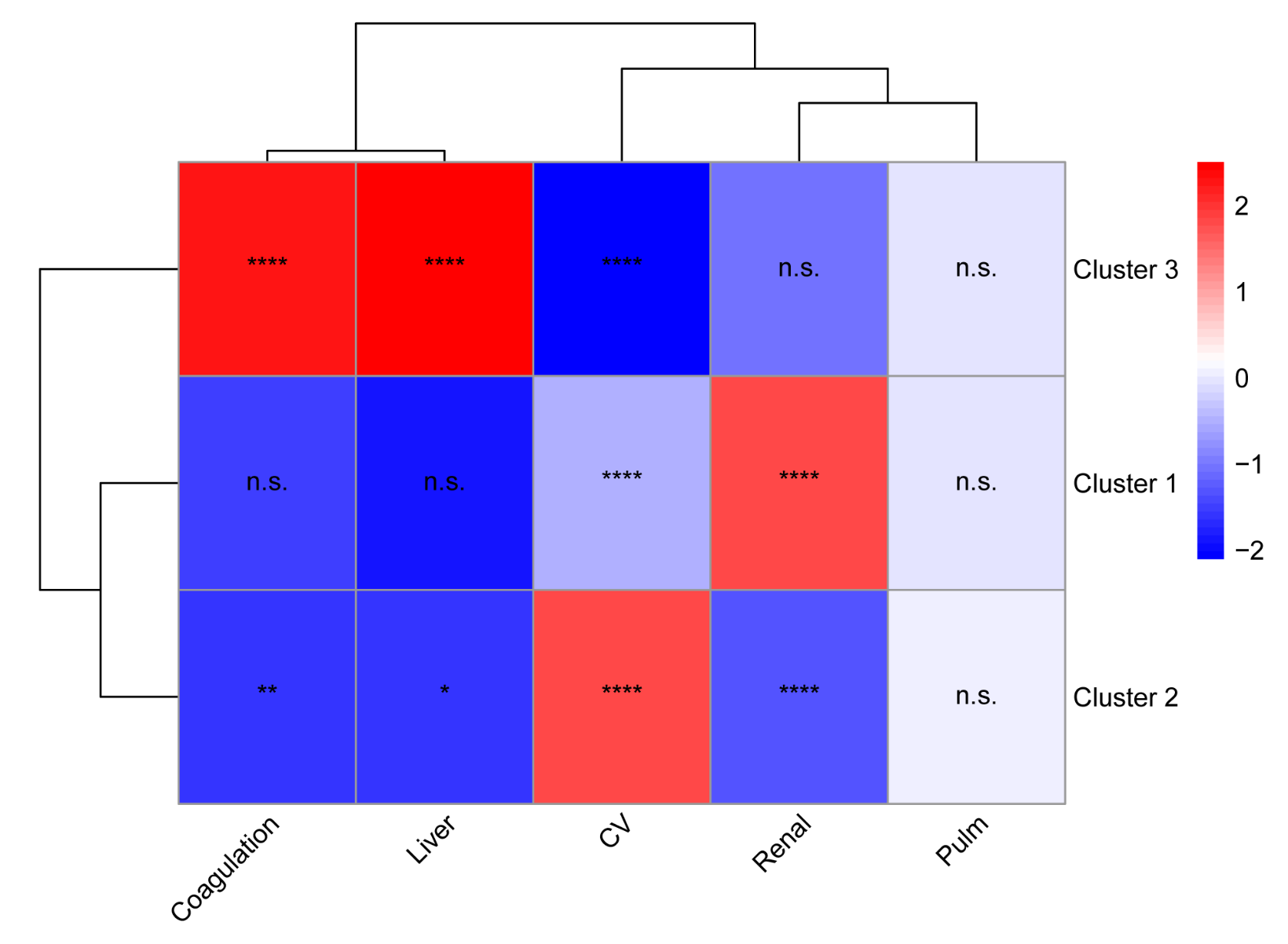


Supplemental Figure 6. Kaplan Meier plot comparing survival time between clusters of ARDS Clinical Trial patients with initial SOFA 9.
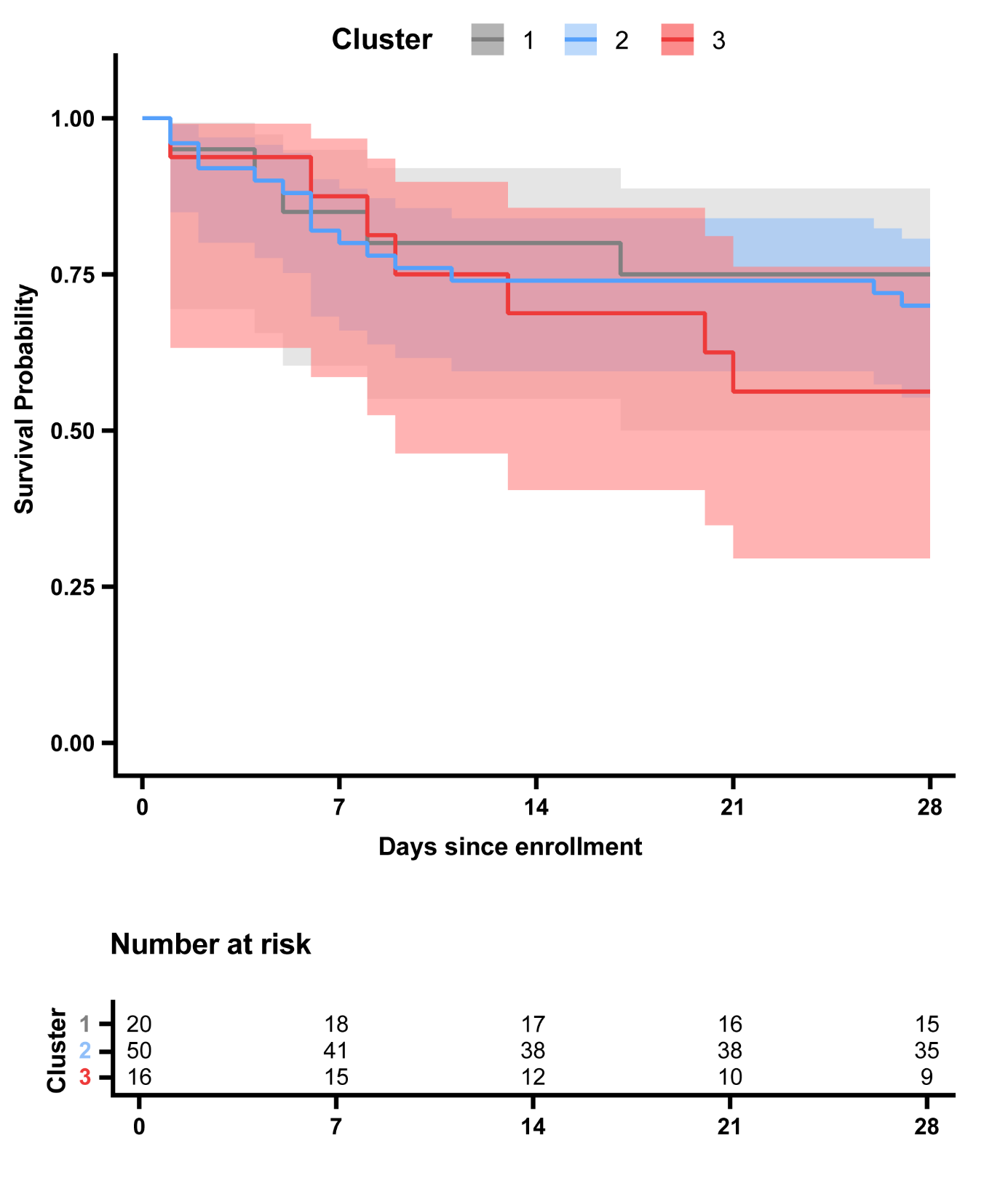


1. Schenck EJ, Hoffman KL, Cusick M, Kabariti J, Sholle ET, Campion TR, Jr. Critical carE Database for Advanced Research (CEDAR): An automated method to support intensive care units with electronic health record data. *J Biomed Inform*. Jun 2021;118:103789. doi:10.1016/j.jbi.2021.103789

2. Schenck EJ, Hoffman KL, Oromendia C, et al. A Comparative Analysis of the Respiratory Subscore of the Sequential Organ Failure Assessment Scoring System. *Annals of the American Thoracic Society*. Nov 2021;18(11):1849-1860. doi:10.1513/AnnalsATS.202004-399OC

3. Rice TW, Wheeler AP, Bernard GR, et al. Comparison of the SpO2/FIO2 ratio and the PaO2/FIO2 ratio in patients with acute lung injury or ARDS. *Chest*. Aug 2007;132(2):410-7. doi:10.1378/chest.07-0617

4. Khanna A, English SW, Wang XS, et al. Angiotensin II for the Treatment of Vasodilatory Shock. *N Engl J Med*. Aug 3 2017;377(5):419-430. doi:10.1056/NEJMoa1704154

5. Singer M, Deutschman CS, Seymour CW, et al. The Third International Consensus Definitions for Sepsis and Septic Shock (Sepsis-3). *JAMA*. Feb 23 2016;315(8):801-10. doi:10.1001/jama.2016.0287

6. Fujii T, Luethi N, Young PJ, et al. Effect of Vitamin C, Hydrocortisone, and Thiamine vs Hydrocortisone Alone on Time Alive and Free of Vasopressor Support Among Patients With Septic Shock: The VITAMINS Randomized Clinical Trial. *JAMA*. Feb 4 2020;323(5):423-431. doi:10.1001/jama.2019.22176

7. National Heart L, Blood Institute PCTN, Moss M, et al. Early Neuromuscular Blockade in the Acute Respiratory Distress Syndrome. *N Engl J Med*. May 23 2019;380(21):1997-2008. doi:10.1056/NEJMoa1901686
